# A Comprehensive Approach: Correlating Ultrasound Imaging with Endometrial Histopathological Analysis in Perimenopausal Women with Heavy Menstrual Bleeding

**DOI:** 10.7759/cureus.57201

**Published:** 2024-03-29

**Authors:** Vidhya Selvam, Pooja Lakshminarayanan

**Affiliations:** 1 Department of Obstetrics and Gynaecology, Sree Balaji Medical College and Hospital, Chennai, IND

**Keywords:** secretory endometrium, disordered proliferative endometrium, proliferative endometrium, endometrial carcinoma, endometrial thickness, atypical endometrial hyperplasia, endometrial hyperplasia without atypia, heavy menstrual bleeding

## Abstract

Introduction: Abnormal uterine bleeding (AUB) is a common troublesome symptom in the perimenopausal age group. The most common type of AUB in this age group is heavy menstrual bleeding. There is a risk of endometrial carcinoma and atypical endometrial hyperplasia in women with AUB in the age group of 40-50 years. Hence early evaluation is of paramount importance in managing women with perimenopausal heavy menstrual bleeding. The current study was undertaken to study the correlation between ultrasound findings and various benign and malignant endometrial histologies in perimenopausal women with heavy menstrual bleeding.

Methodology: Women aged 40-55 years presenting with heavy menstrual bleeding at the gynaecology outpatient department at Sree Balaji Medical College and Hospital, Chennai, India, were included in the study. Patients on anti-platelet and anti-coagulation therapy and patients already on hormonal treatment for heavy menstrual bleeding were excluded from the study. The demographic factors, symptom profiles, ultrasound findings, and histopathological reports were tabulated and analysed.

Results: Of the 147 women included in the study, 75 (51%) were aged 45-50 years and 107 (73%) had two or more pregnancies. Fibroid was the common non-endometrial cause of heavy menstrual bleeding in 52 (35%) cases. The proliferative pattern was the most common non-pathological histology identified in 46 (31%) cases. Endometrial hyperplasia without atypia was the most common pathological histology observed in the study population. Endometrial thickness of more than 8 mm was strongly associated with premalignant or malignant endometrial lesions.

Conclusion: Our study has attempted to identify the correlation between ultrasound evaluation of perimenopausal women with heavy menstrual bleeding and endometrial pathology. Ultrasound, being cost-effective and widely available, is proven to be a tool for first-line investigation of perimenopausal women with heavy menstrual bleeding that guides further evaluation and management.

## Introduction

The World Health Organisation defined perimenopause as the period when endocrinological, biological, and clinical features of menopause begin. For clinical use, perimenopause is a period defined between the onset of menopausal symptoms and one year after menopause. This period signifies the hormonal transition from that of reproductive age to menopause. The average age of menopause in Indian women Is 46±5.59 years [1].

Abnormal uterine bleeding (AUB) is a common troublesome symptom in the perimenopausal age group. AUB in this age group could be in various patterns: heavy bleeding, scanty bleeding, infrequent cycles, frequent cycles, painful cycles, or intermenstrual bleeding. AUB is the cause of visits to a gynaecology clinic in 90% of women in the perimenopausal age group [2]. The most common type of AUB in this age group is heavy menstrual bleeding (HMB) [3]. 

The significance of this symptom in this period increases as it may be the early indicator of endometrial carcinoma [4]. The risk of endometrial carcinoma in women with AUB in the age group of 40-50 years is 0.33 and the combined risk of endometrial carcinoma and atypical endometrial hyperplasia is 1.31 [5]. Hence, early evaluation is of paramount importance in managing women with perimenopausal HMB.

The current study was undertaken to study the correlation between ultrasound findings and various endometrial histologies, both benign and malignant, in women with perimenopausal HMB.

## Materials and methods

This was an analytical study conducted at a tertiary-level teaching hospital, Sree Balaji Medical College and Hospital, Chennai, Tamil Nadu, India. Women aged 40-55 years presenting with HMB at the gynaecology outpatient department of the hospital were included in the study. Patients on antiplatelet and anticoagulation therapy and patients already on hormonal treatment for HMB were excluded from the study. This study was approved by the Institutional Ethical Committee, Sree Balaji Medical College and Hospital (approval number: 002/SBMC/IHEC/2021/1598).

For the purpose fo this study, a normal menstrual cycle was defined as a cycle lasting for two to eight days, with 5-80ml flow every 21-35 days [6]. HMB was defined as a menstrual flow of more than 80 ml [7]. Clinical diagnosis was made on the basis of self-reporting by women of an increase in the number of days of flow and an increase in the number of sanitary pads used [8].

After getting informed consent, history regarding cycle regularity, duration of the heavy flow, obstetric history, previous treatment, and clinical examination findings were noted. Transvaginal ultrasound was done for all participants by experienced radiologists of the hospital. In ultrasound evaluation, endometrial thickness (ET) was measured. The presence of other uterine pathologies namely fibroids, adenomyosis, and endometrial polyp was also noted.

As part of the standard evaluation protocol, all perimenopausal women with HMB underwent endometrial evaluation using a Pipelle endometrial aspirator. Pipelle endometrial sampling is an outpatient procedure. The endometrial samples were sent for histopathological evaluation (HPE). The women got the recommended management according to their histopathological diagnosis. The demographic factors, symptom profiles, ultrasound findings, and histopathological reports were tabulated and analysed using Medcalc statistical software (MedCalc Software Ltd, Ostend, Belgium).

## Results

HMB was most common in the age group of 45-50 years (n=75; 51%) and least common in women belonging to the age group of 40-45 years (n=34; 23%) (Table 1).

**Table 1:** Distribution of HMB across age groups HMB: heavy menstrual bleeding

S.No	Age group (Years)	Number of women with HMB	Percentage
1	40-45	34	23%
2	45-50	75	51%
3	50-55	38	26%

Table 2 gives the distribution of women according to the parity. Among the 147 perimenopausal women included in the study, 107 (73%)had two or more pregnancies while 15 (10%) women were nulli gravida.

**Table 2:** Distribution of HMB across parity HMB: heavy menstrual bleeding

S.No	Parity	Number of women with HMB	Percentage
1	Nulli	15	10%
2	Para 1	25	17%
3	Para 2 and above	107	73%

Perimenopausal women with HMB were divided into three groups based on their ET: (i) ET <8 mm, (ii) ET 8-20 mm, and (iii) ET >20 mm. Of the 147 women, 77 (52%) had ET less than 8 mm. Only 17 (12%) had ET more than 20 mm (Table 3).

**Table 3:** Endometrial thickness in transvaginal ultrasound in women with perimenopausal HMB HMB: heavy menstrual bleeding

S.No	Endometrial thickness	Number of women with HMB	Percentage
1	<8 mm	77	52%
2	8-20 mm	53	36%
3	>20 mm	17	12%

Ultrasound evaluation of the uterus showed two pathologies: fibroid uterus and adenomyosis. The most common non-endometrial pathology detected in our study was fibroid (n=52; 35%). Adenomyosis was present in 13 (9%) women. However, 82 (56%) perimenopausal women did not have any non-endometrial pathology (Table 4).

**Table 4:** Non-endometrial pathologies identified in women with HMB HMB: heavy menstrual bleeding

S.No	Pathologies	Number of women with HMB	Percentage
1	No pathology	82	56%
2	Fibroid	52	35%
3	Adenomyosis	13	9%

Out of the two physiological patterns of the endometrium, the proliferative pattern was the most common in our study (31%) followed by the secretory pattern (27%). Disordered proliferative endometrium, a type of abnormal proliferative endometrium due to unopposed oestrogen action was present in 11% of participants. Another benign pathology observed was endometrial polyp in 8% of participants. Premalignant endometrial lesions, hyperplasia without and with atypia, were observed in 12% and 7% of women, respectively; 4% of women were identified to have endometrial carcinoma (Table 5).

**Table 5:** Endometrial histopathologies in women with HMB HMB: heavy menstrual bleeding; HPE: histopathological examination

S.No	HPE results	Number	Percentage
1	Proliferative endometrium	46	31%
2	Secretory endometrium	39	27%
3	Benign endometrial polyp	12	8%
4	Disordered proliferative endometrium	16	11%
5	Endometrial hyperplasia without atypia	18	12%
6	Endometrial hyperplasia with atypia	10	7%
7	Endometrial carcinoma	6	4%

The distribution of various endometrial histopathological reports is given in Table 6. It is observed that in the group with ET less than 8 mm, normal endometrium (proliferative and secretory) was the most common (86%) and no case of endometrial carcinoma was diagnosed. The most common report in women with ET 8-20 mm was endometrial hyperplasia without atypia (25%) followed by secretory pattern (19%). In the group with ET more than 20 mm, physiological endometrium (proliferative and secretory) was not present. The benign endometrial disease, endometrial polyp, was identified in four (23.5%) women whereas one (6%) woman had disordered proliferative endometrium. The remaining 12 (70.5%) had endometrial hyperplasia/endometrial carcinoma. Out of the women diagnosed with endometrial carcinoma, four had ET more than 20 mm and two had ET 8-20 mm (Table 6).

**Table 6:** Correlation between endometrial histopathology and ET ET: endometrial thickness

S. No	Histopathology	ET <8 mm, n (%)	ET 8-20 mm, n (%)	ET >20 mm, n (%)
1	Proliferative endometrium	37(49%)	9 (17%)	0 (0%)
2	Secretory endometrium	29 (38%)	10 (19%)	0 (0%)
3	Endometrial polyp	1 (0.01%)	7 (13%)	4 (23.5%)
4	Disordered proliferative endometrium	9 (12%)	6 (11%)	1 (6%)
5	Endometrial hyperplasia without atypia	1 (0.01%)	13 (25%)	4 (23.5%)
6	Endometrial hyperplasia with atypia	0 (0%)	6 (11%)	4 (23.5%)
7	Endometrial carcinoma	0 (0%)	2 (4%)	4 (23.5%)

Table 7 analyses the efficacy of the ET cut-off of 8 mm in identifying the premalignant and malignant lesions of the endometrium. Of the 70 women with ET above 8 mm, 33 were found to be having premalignant and malignant pathologies. This is found to be statistically significant (P value <0.05). The sensitivity of ET >8 mm in predicting abnormal pathology is 97% and the specificity is 67.2% (Table 7).

**Table 7:** Association of endometrial thickness >8 mm with premalignant and malignant endometrial pathologies ET: endometrial thickness

S.No	Histopathology	ET <8 mm, n	ET >8 mm, n
1	Benign	76	37
2	Premalignant and malignant	1	33

The association of ET more than 20 mm with premalignant and malignant lesions was analysed. Table 8 shows that ET of more than 20 mm is strongly associated with premalignant and malignant lesions of the endometrium (P value <0.05). The specificity of ET >20mm in predicting premalignant and malignant lesions is 95.5% and sensitivity is 70% (Table 8). 

**Table 8:** Association of ET >20 mm with premalignant and malignant endometrial pathologies ET: endometrial thickness

S.No	Endometrial thickness	Benign, n	Premalignant and malignant, n
1	ET <20 mm	108	22
2	ET >20 mm	5	12

## Discussion

Perimenopause is the period of depleting ovarian function. Due to frequent anovulation, the endometrium is subjected to unopposed estrogen action. Because of the stimulatory effect of estrogen, endometrial hyperplasia and endometrial carcinoma can develop [9]. In our study, out of 147 perimenopausal women with HMB, 75 (51%) belonged to the age group of 45-50 years (Table 1). This is in concurrence with the study by Baghel et al. [3]. The mean age of women with perimenopausal AUB was 44.77±4.47 years in the study by Mayuri et al. [10]. Among our study sample, 107 (73%) were second gravida and above and 15 (10%) were nulligravida (Table 2). This parity distribution was also observed by Baghel et al., where 76% were second gravida and more [3]. 

In our study, 35% of women had fibroid on ultrasound evaluation (Table 3). This is similar to the finding in the study conducted by Daga et al., where 39.4% of women had fibroid [11]. Another benign condition of the uterus associated with HMB is adenomyosis. In our study, 9% of women had adenomyosis. This is less than that observed in the study by Daga et al. (15.4%) [11]. 

In our study, 17 (12%) women had ET above 20 mm, 53 (36%) had between 8-20 mm and 77(52%) had ET less than 8 mm (Table 4). This is similar to the study by Jaiswal et al., where 45% of women had ET less than 9 mm [12]. In contrast, only 19% of women had ET less than 8 mm in a study by Mathew et al. [13] and 29% in a study by Sah et al. [14]. In our study, ET more than 20 mm was observed in 10.2% of women, similar to the study by Pillai [15].

In our study, a proliferative pattern was the most common histology (31%), followed by a secretory pattern (22%). This is similar to the study by Manjari et al., which reported a higher incidence of proliferative pattern (40%) in the perimenopausal age group [16]. The high incidence of the proliferative pattern could be explained by the high prevalence of anovulatory cycles in the perimenopausal age due to the depletion of ovarian follicles.

In the study by Pillai [15] and Mathew et al. [13], secretory pattern was the most common histology. In the study conducted by Sajitha et al., endometrial hyperplasia was the most common pathology (25%) followed by secretory pattern (16.7%) [17]. Benign pathologies observed in our study were disordered proliferative endometrium and endometrial polyp. In the study by Mathew et al., disordered proliferative endometrium was observed in 29% [13]. The prevalence of endometrial polyps was much varying between the studies. In our study, 8% of women had endometrial polyps. A study by Byna et al. [18] observed endometrial polyps in 3% of women whereas Bhosle et al. [19] observed endometrial polyp in 46.9% women. Endometrial polyps are a benign localized overgrowth of endometrial tissue protruding into the uterine cavity, the pathogenesis of which is unknown. However, they are believed to develop due to unbalanced estrogens and progestin [20].

Endometrial carcinoma was identified in 4% of women, endometrial hyperplasia without atypia was identified in 12%, and atypical hyperplasia was observed in 7%. The prevalence of endometrial carcinoma is similar to that in the study by Getpook and Wattanakumtornkul, where endometrial cancer was observed in 3.6% of women [21]. In the study by Gawron et al., atypical hyperplasia was observed in 1.4%, endometrial hyperplasia without atypia in 7.5%, and endometrial cancer in less than 1% of women [22].

ET less than 8 mm is a good predictor of benign histology. Zero cases of endometrial cancer were reported in women with ET less than 8 mm. In our study, the sensitivity of ET more than 8 mm in predicting premalignant and malignant endometrial pathology was 97% making it a good screening cut-off for endometrial pathologies (Table 7). The study by Getpook and Wattanakumtornkul suggested 8 mm as the cut-off for recommending endometrial sampling for perimenopausal women with HMB, with sensitivity of 83.9% and specificity of 58.8% [21]. Ozendemir et al. also found that ET of 8 mm is a good cut-off for benign histologies with sensitivity of 98.6% and specificity of 56.4% [23]. The study by Mayuri et al. showed the the sensitivity and specificity of transvaginal ultrasound in diagnosing abnormal endometrium was 78.6% and 87.8%, respectively [10].

ET of more than 20 mm is highly specific of endometrial hyperplasia and carcinoma-95.5% (Table 8). In a study conducted by Batra et al., the mean ET in women with endometrial carcinoma was 18 mm [24]. In the same study, the mean ET associated with endometrial hyperplasia was 14 mm. Knowledge regarding the strong association of ET of more than 20 mm with endometrial carcinoma emphasises the significance of counselling the women regarding the importance of further evaluation and planning advanced imaging-MRI, before doing HPE.

The limitation of our study was that the samples were recruited from a single centre and the sample size was small. To generalise the findings of this study to our population, multi-centred studies with larger sample sizes should be conducted. Obesity, polycystic ovary syndrome (PCOS), infertility, and type 2 diabetes mellitus are some of the known risk factors for endometrial carcinoma. In future studies, collecting data regarding the presence of these factors, doing subgroup analysis, and developing scoring systems are recommended to predict the risk of endometrial carcinoma/hyperplasia at a particular endometrial thickness that would guide the effective management of perimenopausal women with HMB.

## Conclusions

Ultrasound, being cost-effective and widely available, serves as a tool for first-line investigation of perimenopausal women with HMB that guides further evaluation and management. The present study undertaken to identify the correlation between ultrasound evaluation of perimenopausal women with HMB and endometrial histopathologies showed that benign histopathologies are more common than endometrial carcinoma and hyperplasia in this group. It showed that ET less than 8 mm serves as a good predictor of normal endometrium and ET more than 20 mm as a strong predictor of premalignant and malignant endometrial pathologies. This has to be validated by further multicentred studies with large sample sizes. 
